# Genome-Wide Assessment of Runs of Homozygosity and Estimates of Genomic Inbreeding in a Chinese Composite Pig Breed

**DOI:** 10.3389/fgene.2021.720081

**Published:** 2021-09-01

**Authors:** Zhong Xu, Shuqi Mei, Jiawei Zhou, Yu Zhang, Mu Qiao, Hua Sun, Zipeng Li, Lianghua Li, Binke Dong, Favour Oluwapelumi Oyelami, Junjing Wu, Xianwen Peng

**Affiliations:** ^1^Hubei Key Laboratory of Animal Embryo and Molecular Breeding, Institute of Animal Husbandry and Veterinary, Hubei Provincial Academy of Agricultural Sciences, Wuhan, China; ^2^Department of Animal Science, School of Agriculture and Biology, Shanghai Jiao Tong University, Shanghai, China

**Keywords:** pig, crossbreed, runs of homozygosity, inbreeding, linkage disequilibrium

## Abstract

The primary purpose of the current study was to assess the genetic diversity, runs of homozygosity (ROH) and ROH islands in a Chinese composite pig and explore hotspot regions for traces of selection. First, we estimated the length, number, and frequency of ROH in 262 Xidu black pigs using the Porcine SNP50 BeadChip and compared the estimates of inbreeding coefficients, which were calculated based on ROHs (F_ROH_) and homozygosity (F_HOM_). Our result shows that a total of 7,248 ROH exceeding 1Mb were detected in 262 pigs. In addition, *Sus scrofa* chromosome (SSC) 8 and SSC10, respectively, has the highest and lowest chromosome coverage by ROH. These results suggest that inbreeding estimation based on total ROH may be a useful method, especially for crossbreed or composite populations. We also calculated an inbreeding coefficient of 0.077 from the total ROH. Eight ROH islands were found in this study. These ROH islands harbored genes associated with fat deposition, muscular development, reproduction, ear shape, and adaptation, such as *TRAF7*, *IGFBP7*, *XPO1*, *SLC26A8*, *PPARD*, and *OR1F1*. These findings may help to understand the effects of environmental and artificial selection on the genome structure of composite pigs. Our results provide a basis for subsequent genomic selection (GS), and provides a reference for the hybrid utilization of other pig breeds.

## Introduction

Crossbreeding is a common strategy to improve livestock production because it can explore complementarities of additive genetic effects as well as heterosis caused by non-additive genetic effects (Howard et al., 2016). The Enshi black pig, a typical native black breed in China, mainly lives in mountainous areas of southwest China at an average altitude of more than 800m. It is well-known for its adaptability to a mountainous environment, excellent meat quality, fat storage ability, and cold-wet tolerance (China National Commission of Animal Genetic Resources, 2011). Since the 1990s, under the impact of exotic germplasm with high growth rate and lean meat rate, the Enshi black pig has been facing extinction due to its low growth rate. To overcome these deficiencies and conserve the Enshi black pig, crossbreeding programs have been implemented to increase productivity, and the Xidu black pig is a new composite breed that has been developed for this situation. Crossbreeding combines the cold-wet environmental adaptation of the Enshi black pig with the high fertility of the Meishan pig and the fast growth rate of the Hubei white pig. When the three-way crossbreeding [Hubei white×(Meishan×Enshi black)] was formed, they were inter-se mating and selected to become the Xidu black pig breed, which having about 50% Hubei white, 25% Meishan, and 25% Enshi black pig inheritance. It is worth noting that the Xidu black pig is now well established and can be used as a purebred without the need for any ongoing crossbreeding programs.

Generally, crossbred offspring can be mated among themselves in each generation, and selection for specific traits and genetic improvement can be applied during this process. Therefore, it is essential to manage genetic diversity by avoiding high inbreeding rates in composite breed, which will retain high levels of heterozygosity and heterosis (Peripolli et al., 2020). It is difficult to assess genetic diversity using pedigree data mostly because the genealogical relationships between parental breeds that are used in crossbreeding cannot be established. However, the analysis of genomic data could solve this problem (Ganteil et al., 2020). There are several methods used in estimating genetic diversity from genomic data. Some of these methods include the use of observed and expected heterozygosity, runs of homozygosity (ROH), and linkage disequilibrium (LD).

Runs of homozygosity are long continuous homozygous segments in the genome that are formed in an individual by the combination of two identical haplotypes from a common ancestor (Ceballos et al., 2018). As an important genome feature, ROH provides an essential reference for the study of the genome structure. Besides, in animal genetics, the presence of homozygous segments in the genome can be influenced by intensive selection, population history, and consanguinity levels (Peripolli et al., 2017). Also, inbreeding estimates based on ROH is usually considered to be more accurate for estimating individual inbreeding levels when compared with other existing methods (Keller et al., 2011). In addition, ROH hotspots are known to be non-randomly distributed across the genome, and can reveal selection pressure events since selection is one of the main causes of homozygous stretches on the genome. Recently, ROH has been mostly employed in estimating the genomic inbreeding and selection signatures of many livestock populations (Zhang et al., 2018b; Xu et al., 2019; Shi et al., 2020), but less commonly used in crossbred or composite populations.

The LD analysis is also an efficient approach for determining the level of genetic diversity within a studied population. Generally, LD can be defined as the non-random genetic relationship between two loci in a population (Saravanan et al., 2020). Thus, exploring the pattern and extent of LD in the genome can provide essential insights for guiding genome-wide association studies (GWAS) and genome selection (GS; Mokry et al., 2014).

The main objectives of this study were: (1) to investigate the characteristics of ROH on the genome of the Xidu black pig, and also identify the genomic regions with high ROH frequency; and (2) to estimate genetic diversity parameters, such as inbreeding rates and LD in this composite pig population.

## Materials and Methods

### Ethical Statement

All experimental procedures were approved by the Institutional Animal Care and Use Committee of the Hubei Academy of Agriculture Sciences, and all methods involved pigs were in accordance with the agreement of Institutional Animal Care and Use Committee of the Hubei Academy of Agriculture Sciences (Permit number: 36/2016).

### Sample Collection, SNP Genotyping, and Quality Control

The animal genomics dataset used in the current study were gotten from pigs raised in a composite swine breeding farm located in Enshi, Hubei, China. In order to make the sample representative, we chose individuals from the core group to avoid full siblings. At last, there were 262 individuals consisting of approximately three generations. The 262 animals were genotyped using the Porcine Single nucleotide polymorphism SNP50 BeadChip (Illumina, United States), consisting of 51,315 Single nucleotide polymorphism (SNPs) evenly distributed along the pig genome. Genotype quality control was carried out using PLINK v1.90 (Chang et al., 2015) software based on the following filtering criteria: (1) the call rate of SNPs and individuals were higher than 0.9; (2) the minor allele frequency (MAF) was greater than 0.01; and (3) only SNPs mapped to autosomes were included. The latest version of the pig genome, *Sus scrofa* 11.1 was used in this study.

### Runs of Homozygosity Detection and Classification

We identified ROH in individuals using PLINK v1.90 software, which uses a sliding window approach to detect autozygous segments. The algorithm is as follows: take a window of X SNPs and slide them across the genome. Determine at each window position whether the window looks sufficiently “homogeneous” (yes/no). Then, for each SNP, calculate the proportion of the “homozygous” window that overlaps that position. Call segmentation based on this metric, such as a threshold based on the average value. To define a ROH, the criterion and thresholds were as follows: (1) a minimum ROH length of 1Mb; (2) at least 50 homozygous SNPs included in a ROH; (3) a minimum density of a SNP in 100Kb; (4) a sliding window of 50 SNPs across the genome that moves one SNP at a time; and (5) up to one heterozygous SNP and five missing SNPs were allowed in a sliding window. Detected ROHs were later classified into three different classes based on their length: 1–5, 5–10, and >10Mb. The total number and length of ROHs were counted for all individuals.

### Detection of Common Runs of Homozygosity and Gene Annotation

We identified the genomic regions that were mostly associated with ROHs, by calculating the proportion of SNPs in ROH. This was done by counting the number of times the SNP was detected in those ROH across individuals. Afterward, we selected the top 1% of SNPs that were commonly observed in ROHs. Adjacent SNPs that were above this threshold were finally merged into genomic regions which are called ROH islands, which is characterized by being shared by a majority of individuals in the population (Dixit et al., 2020). We used the database provided by NCBI to annotate the genes in the ROH island. Through a large number of accurate literature searches, the biological function of each annotated gene in the ROH island was inferred.

### Estimation of Genomic Inbreeding Coefficient

In this study, two types of genomic inbreeding coefficients were calculated, one based on ROH (*F_ROH_*) and the other based on excess of homozygosity (*F_HOM_*). Genomic inbreeding coefficients (*F_ROH_*) were computed for all individuals by the following formula, as proposed by McQuillan et al. (2008):

$$F_{ROH}=\frac{\sum L_{ROH}}{L_{auto}},$$

where *ΣL_ROH_* is the length of ROHs, and *L_auto_* is the total length of the genome covered by the SNPs included in this chip. *F_ROH_* was also calculated based on three length classes: 1–5, 5–10, >10, and total (>1) Mb. Genomic inbreeding coefficients (*F_HOM_*) were calculated as $F_{HOM}=\frac{O-E}{L-E}$, where *O* is the number of observed homozygous genotypes, *E* is the number of expected homozygous genotypes by chance, and *L* is the total number of genotyped autosomal SNPs. Pearson’s correlation was used to compare the inbreeding coefficients estimated by these methods using R.

### Extent of Linkage Disequilibrium

The LD was measured using the *r*^2^, which was calculated for each pair of SNPs per chromosome according to Hill and Robertson (1968). The pairwise LD (*r*^2^) were calculated using the parameters “--ld-window 99,999 --ld-window-kb 1,000 --ld-window-r2 0” in PLINK v1.90. To visualize the decline of LD, the physical distances between SNPs were divided into 100-Kb intervals, and the average of *r*^2^ in each group was then estimated.

## Results and Discussion

After quality control, 262 pigs and 38,275 SNPs were retained. The average observed (*H_o_*) and expected (*H_e_*) heterozygosity estimates were 0.37 and 0.35, respectively, and the average MAF was 0.26. We observed that the *H_o_* was somewhat higher than the *H_e_*.

### Distribution of ROH

In this study, a total of 7,248 ROHs were identified in the 262 animals with an average of 27.66 ROH per animal. The average ROH length was 6.32Mb, and the longest fragment in *Sus scrofa* chromosome 8 (SSC8) was 57.84Mb (1,000 SNPs). Table 1 summarizes the descriptive statistics of the ROH number and length by classes. The total ROH number of the composite Xidu Black pigs was mainly composed of shorter segments (1–5Mb), which accounted for about 56.16% of all the detected ROH. The genome coverage of long segments (>10Mb) accounted for 37.11% of the total ROH. Short ROH reflects ancestral inbreeding history, while long ROH segments are usually formed by recent inbreeding. This indicated that both ancient and recent inbreeding events might have affected this population, but recent inbreeding or selection pressures have mainly influenced the genome of the Xidu Black pig population.

**Table 1:** Descriptive statistics of runs of homozygosity (ROH) number and length (in Mb) by ROH length class (ROH 1–5Mb, ROH 5–10Mb, ROH>10Mb, and total).

ROH length (Mb)	ROH number	Percent (%)	Mean length (Mb)	Genome coverage (%)
1–5	4,072	56.18	3.49	31.02
5–10	2,134	29.44	6.84	31.88
>10	1,042	14.38	16.32	37.11
Total (>1)	7,248	100	6.32	100

**Figure 1:**
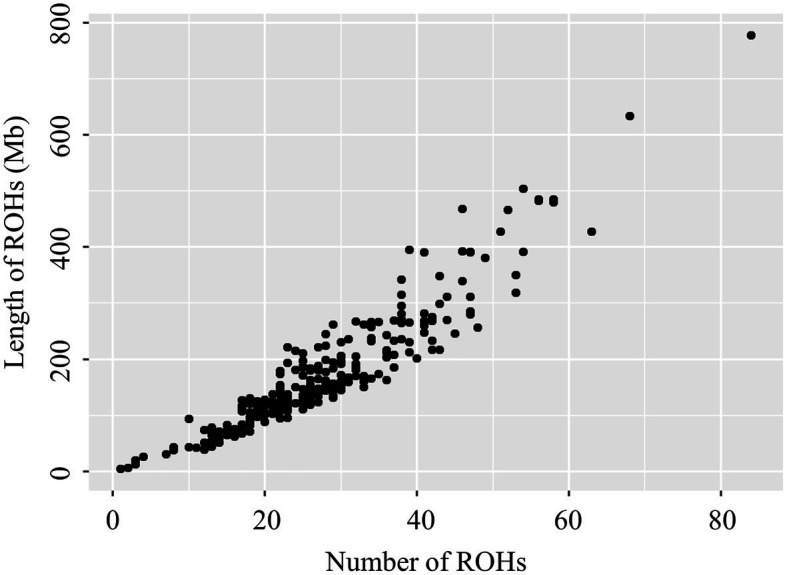
Relationship between the total number of ROH segments (*x*-axis) and the total length (Mb) of the genome in ROH (*y*-axis) for all individuals. Each dot represents an individual.

The relationship between the total ROH number and the total length of the genome covered by ROH in each individual is shown in Figure 1, and is greatly different among animals. In this population, the most extreme animal with long ROHs had a length of 777.06Mb (34.41% of the pig genome). The variability of the total number and length of ROH among individuals was high. Similar distributions were also observed in other pigs (Xu et al., 2019; Shi et al., 2020) and livestock species, such as sheep (Mastrangelo et al., 2017) and cattle (Peripolli et al., 2018).

**Figure 2:**
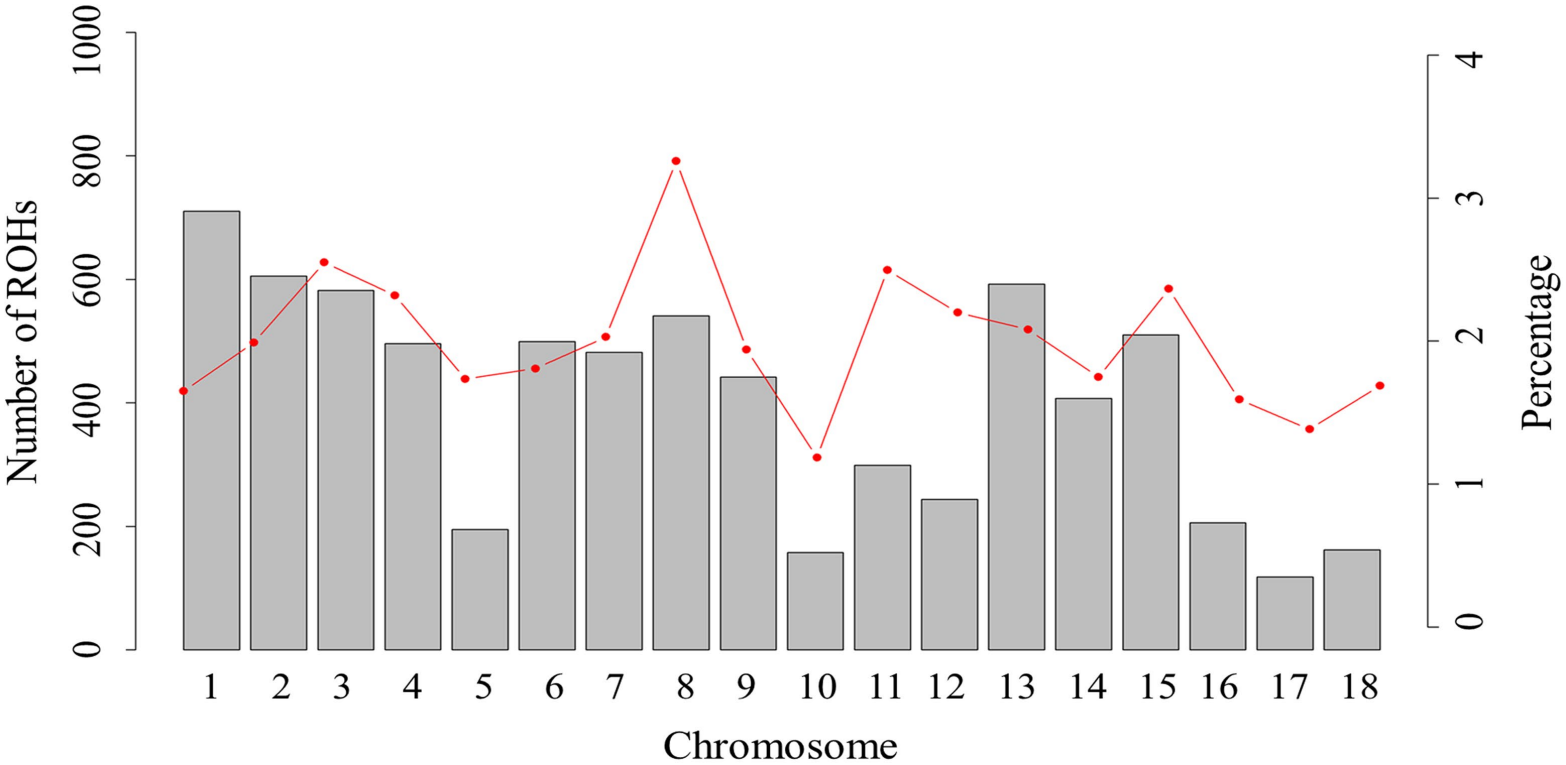
The number of ROHs and percentage coverage per chromosome in Xiduhei pig population. The vertical bars show the total number of ROHs per chromosome and the line shows the percentage of chromosome covered with ROH.

For chromosomes, the distribution of total number of ROHs in each chromosome and percentage coverage per chromosome are presented in Figure 2. The number of ROHs per chromosome was greater on SSC1 (710 segments), while the smallest number of ROHs was on SSC17 (118 segments). Previous studies on pigs (Xu et al., 2019; Zhan et al., 2020) have also reported the highest number of ROH on SSC1, possibly because SSC1 is the largest chromosome in the pig genome, and has more markers than other chromosomes. The highest ROH coverage was observed on SSC8 (3.26%), whereas the lowest was on SSC10 (1.19%). Our result suggests that the chromosomes with high ROH coverage might have been influenced by positive selection, which consequently increases the accumulation of advantageous alleles on the chromosome. According to our results, some genomic regions, with the highest ROH coverage, on the SSC4 require more consideration.

### Genomic Inbreeding Coefficients

Here, we calculated the genomic inbreeding coefficients (*F_ROH_* and *F_HOM_*) using the genotype data of 262 individuals. The mean value of the inbreeding coefficient based on total observed ROHs (F_ROH_total_) was 0.077 and ranged from 0.002 to 0.344. The estimated F_HOM_ inbreeding coefficients was −0.054 with a range from −0.199 to 0.251 in this population (negative values correspond to individuals with lower-than-average homozygosity). Traditionally, the inbreeding coefficient was estimated based on pedigree data. However, when it comes to cross-bred individuals, things get complicated because the genealogical relationships between parental breeds cannot be established. Moreover, in reality, pedigree information might be incorrect and incomplete, and does not usually take into account the various stochastic events of recombination, which might have occurred during meiosis (Marras et al., 2015). Thus, the inbreeding coefficient value estimated based on pedigree data could not totally show the actual relatedness among individuals within a population. In this study, we used genomic data to estimate inbreeding coefficient.

**Figure 3:**
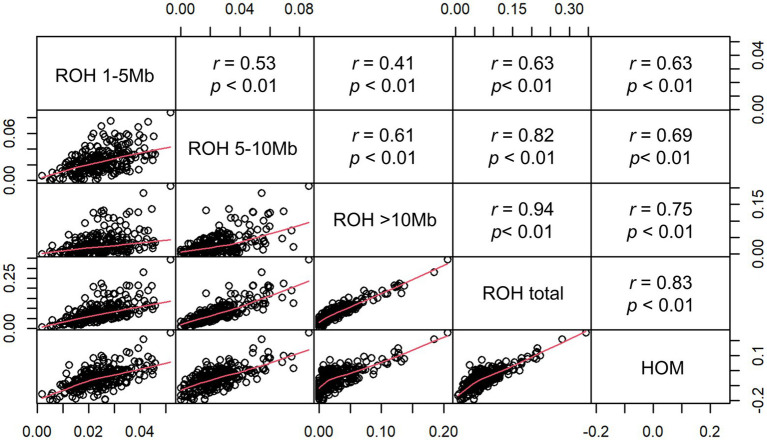
Scatterplots (lower panel) and Pearson’s correlations (upper panel) of the genomic inbreeding coefficients F_ROH_ (F_ROH 1~5 Mb_, F_ROH5~10 Mb_, F_ROH>10 Mb_, and F_ROH_total_) and F_HOM._

To further investigate the inbreeding coefficients which were obtained by different estimation methods, we conducted a pairwise comparisons between F_HOM_ and F_ROH_. The pairwise correlations among five types of inbreeding coefficients were shown in Figure 3. Among all pairwise correlations, the highest correlation was 0.94 between F_ROH_total_ and F_ROH>10Mb_. This result showed that long ROH segments (>10Mb) were the main source of F_ROH_total_. The inbreeding coefficients obtained by different categories of ROHs with F_HOM_ ranged from 0.63 to 0.83, with the highest correlation found between F_ROH_total_ and F_HOM._ These results are in line with previous research in other pig populations (Xu et al., 2019; Shi et al., 2020) and cattle (Mastrangelo et al., 2016; Biscarini et al., 2020). Furthermore, we found that many individuals had a negative F_HOM_ value, which might be because F_HOM_ was sensitive to allele frequency for populations with a higher level of heterozygosity compared to F_ROH_ estimators (Zhang et al., 2015). Zhang et al. (2015) found a negative F_HOM_ value for Danish Red Cattle (RDC), a composite breed, which is likely due to the admixture present in RDC. A similar result was obtained for a crossbred cattle in Vrindavani (Chhotaray et al., 2021). Therefore, the results of this study suggest that the inbreeding level based on total ROH may be a useful method, especially for crossbreed or composed populations.

### ROH Islands

**Figure 4:**
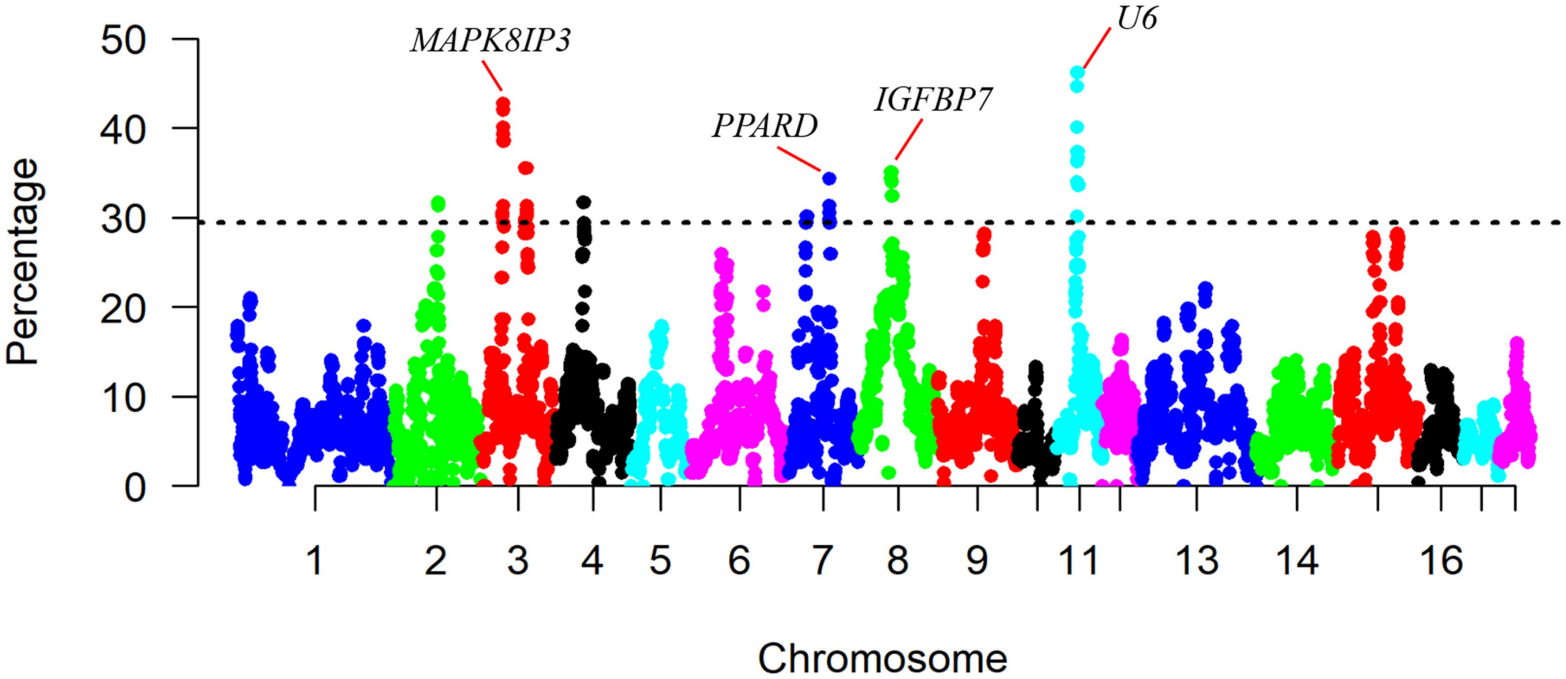
Manhattan plot of occurrences (%) of a SNP in ROHs across individuals.

Furthermore, we plotted the percentage of SNPs in ROHs against their respective positions along the chromosomes (Figure 4). The result shows a non-uniformity in the frequency of different SNPs within the ROH regions across the genome. The most frequent SNP in ROH (121 occurrences, 46.18%) was mapped at ∼35Mb on SSC11, and the closest gene to this SNP was the *U6* gene. Regions of the genome with high homozygosity around the ROH islands may contain positively selected targets and might be under strong selection pressure (Pemberton et al., 2012). To identify the genomic regions that were mostly associated with ROH in all individuals, we considered the top 1% of SNPs with the highest occurrences (over 30.15% of the samples) in the ROH as candidate SNPs (Figure 4). We identified a total of eight ROH island regions (Table 2), with length ranging from 0.804Mb on SSC2 to 3.188Mb on SSC11.

**Table 2:** List of genomic regions of extended homozygosity detected in Xidu black pigs.

CHR	Start (bp)	End (bp)	Length (bp)	SNPs	Genes
2	77,541,872	78,345,614	803,743	19	22
3	38,431,596	41,463,322	3,031,727	82	123
3	78,653,188	81,780,864	3,127,677	62	18
4	47,685,504	48,592,012	906,509	26	2
7	31,217,540	32,755,132	1,537,593	30	26
7	70,752,052	71,626,349	874,298	20	1
8	56,064,681	58,992,457	2,927,777	77	3
11	34,189,314	37,377,736	3,188,423	66	2

Chromosome position, the start and end position of ROH, ROH length, number of SNPs, and the number of genes within the ROH islands were reported in Table 2. To evaluate the potential functional importance of the detected ROH islands, we analyzed the gene content of the identified regions. In summary, we annotated a total of 199 genes that were detected within these ROH islands. The chromosome position, start and end, gene name and Ensembl Gene ID were provided in Supplementary Table S1.

### Candidate Genes Within Runs of Homozygosity Islands

In this study, we focus on the genes related to some specific livestock-traits which are also important in breeding. We identified numerous candidate genes associated with muscular development and fat deposition. Among these genes is the *TRAF7* gene, a MyoD1 transcriptional target that can regulate NF-κB activity during myogenesis. Studies have shown that missense mutations in *TRAF7* causes developmental delay or skeletal dysplasia (Tsikitis et al., 2010; Tokita et al., 2018). *IGFBP7* can promote lipid accumulation and triglyceride production in mature adipocytes and plays an important regulatory role in the differentiation of preadipocyte cells that can affect fat deposition (Hu et al., 2021). *PRSS33* was related to lipid transport and metabolism, and was also detected in reported selection signature regions in Enshi black pigs, one of the founder breeds. Since Xidu black pigs are characterized for meat quality, we also detected some important genes that are associated with specific meat quality traits: *TRAP1* and *CREBBP* are associated with pork meat pH in Finnish Yorkshire pigs (Verardo et al., 2017); *SLC9A3R2* gene has been shown to be differentially expressed in longissimus muscle tissues of Meishan and Large White pigs (Jiugang et al., 2011), and was considered to be the candidate genes for meat quality (Wu et al., 2020). *OTX1* is a novel regulator of proliferation, migration, invasion, and apoptosis in lung adenocarcinoma (Yang et al., 2020), and were involved in the battle between foot-and-mouth disease virus and the host (Zhang et al., 2018a). We identified several candidate genes related to reproduction traits: *XPO1*, a nuclear transport receptor, plays an essential role in meiotic resumption in porcine full-grown and growing oocytes (Onuma et al., 2018); *SLC26A8*, also known as testis anion transporter 1, is required for sperm terminal differentiation and male fertility in the mouse (Toure et al., 2007).

*OR1F1*, an olfactory receptor, was demonstrated to function in odor perception activation (Li et al., 2013). *CLDN9* played an essential role in maintaining barrier function in airway epithelial cells (Gon et al., 2017), and *E4F1* is essential for skin homeostasis (Lacroix et al., 2010). Notably, the Xidu black pigs reside in a subtropical region, which is characterized by high temperature and humidity. Therefore, we considered that *OR1F1*, *CLDN9*, and *E4F1* as key factors for the environmental adaptability of Xidu black pigs. The most interesting candidate gene in this population seems to be *PPARD* gene, which was shown to affect the shape of the external ear and fat deposition in pigs (Meidtner et al., 2009; Ren et al., 2011). Due to the hybrid parents (Hubei white pig, Meishan pig, and Enshi black pig) have different ear shapes, the ear shapes of the base population had erected, forward sloping and drooping phenotype. Therefore, during the breeding process of Xidu black pigs, the forward sloping and slightly drooping ear shape was constantly selected. This gene may play a key role in ear shape in this population and needs more attention.

### Linkage Disequilibrium

**Figure 5:**
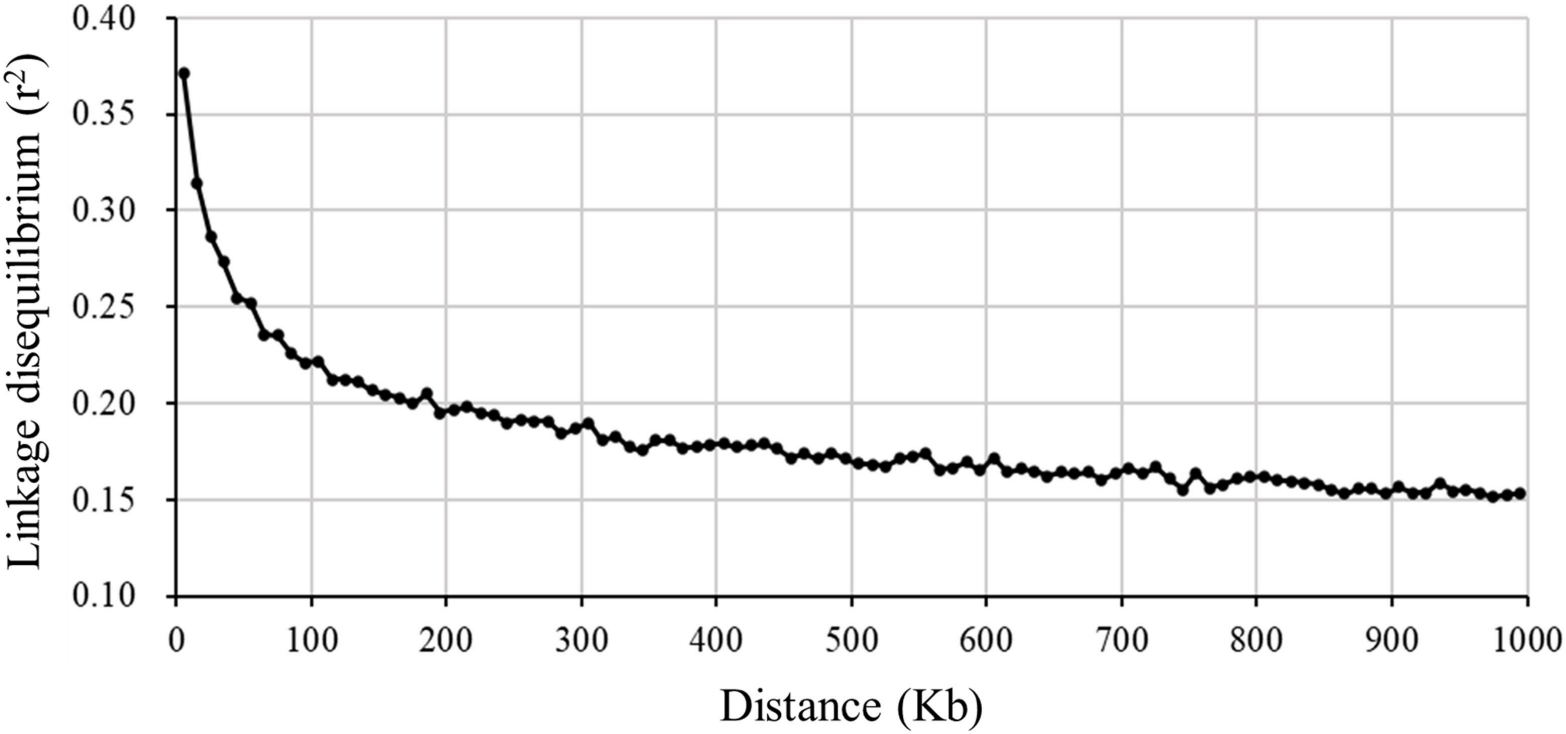
Decay of average linkage disequilibrium (*r*^2^) over distance (Kb) between markers for a composite pig breed.

A total of 38,275 SNPs were used to calculate the average LD between all adjacent SNPs, with a distance less than 100Kb. The average and SD of estimated *r*^2^ was 0.289±0.316, which was similar to the result that (Joaquim et al., 2019) estimated in a crossbred Landrace pig population. It was observed that the LD value decreased with the increase of the distance between markers (Figure 5). When the distance was greater than 1,000Kb, the average *r*^2^ was about 0.15. The LD extent (*r*^2^=0.3) in Xidu black pigs was about 25Kb. This value was less than 79.54Kb (*r*^2^=0.3) for Meishan pigs (one of the original parental breed) obtained by the same method (Liu et al., 2020). The effectiveness of GWAS and genomic selection (GS) relies on the LD between the markers. According to the literature, a mean *r*^2^ value above 0.30 is considered as a strong LD sufficient for QTL mapping (Shifman et al., 2003). However, an average *r*^2^ of 0.20 is considered enough to achieve an accuracy of 0.85 for genomic estimated breeding value (GEBV; Meuwissen et al., 2001). In this study, we found that moderate LD (*r*^2^≥0.2) extended up to 150–200Kb. Assuming that the total length of the pig genome is 2.5Gb, this suggests that at least 12,500–16,667SNPs would be required for effective GWAS in this breed. This result could be particularly useful when designing breed-specific SNP array panels in future genomic study and selection programs for this composite pig breed.

## Conclusion

In summary, in this study, we investigated the patterns of ROH, inbreeding coefficients and LD in the Xidu black pigs. To our knowledge, this is the first study about ROH patterns and autozygosity islands in a composite pig breed. The results of this study suggest that inbreeding based on total ROH may be a useful method, especially for crossbred or composite populations. The detected ROH patterns in this population suggests recent inbreeding events, agreeing with the newest developments in this composite pig breed. Besides, the reported genes within the identified ROH islands point to phenotypic characteristics related to reproduction, fat deposition, ear shape, and environmental adaptation. We believe that these findings will further assist in genome-wide association studies, GS, as well as the design and implementation of breed improvement and conservation programs.

## Data Availability Statement

The datasets presented in this study can be found in online repositories. The names of the repository/repositories and accession number(s) can be found at: https://figshare.com/articles/dataset/Genome-wide_assessment_of_runs_of_homozygosity_and_estimates_of_genomic_inbreeding_in_a_Chinese_composite_pig_breed/14904567.

## Ethics Statement

All experimental procedures were approved by the Institutional Animal Care and Use Committee of the Hubei Academy of Agriculture Sciences, and all methods involved pigs were in accordance with the agreement of Institutional Animal Care and Use Committee of the Hubei Academy of Agriculture Sciences (Permit number: 36/2016).

## Author Contributions

SM and XP: data curation and supervision. SM: funding acquisition. JW: project administration. ZX: writing – original draft. SM, XP, and FO: writing – review and editing. JZ, YZ, MQ, HS, ZL, LL, and BD: data analysis. All authors contributed to the article and approved the submitted version.

## Conflict of Interest

The authors declare that the research was conducted in the absence of any commercial or financial relationships that could be construed as a potential conflict of interest.

## Publisher’s Note

All claims expressed in this article are solely those of the authors and do not necessarily represent those of their affiliated organizations, or those of the publisher, the editors and the reviewers. Any product that may be evaluated in this article, or claim that may be made by its manufacturer, is not guaranteed or endorsed by the publisher.
