# Supplementary material for: Genome-Wide Assessment of Runs of Homozygosity and Estimates of Genomic Inbreeding in a Chinese Composite Pig Breed
Source: Front Genet. 2021 Sep 1;12:720081. doi: 10.3389/fgene.2021.720081 (PMC8440853; doi:10.3389/fgene.2021.720081)
Supplement: Supplementary file 1 [file Table_1.DOCX]

**Genome-wide assessment of runs of homozygosity and estimates of genomic inbreeding in a Chinese composite pig breed**

Zhong Xu^1^, Shuqi Mei^1^, Jiawei Zhou^1^, Yu Zhang^1^, Mu Qiao^1^, Hua Sun^1^, Zipeng Li^1^, Lianghua Li^1^, Binke Dong^1^, Favour Oluwapelumi Oyelami^2^, Junjing Wu^1§^, Xianwen Peng^1§^

Running title: Genome ROH in composite pig breed

^1^Hubei Key Laboratory of Animal Embryo and Molecular Breeding, Institute of Animal Husbandry and Veterinary, , Wuhan 430064, China

^2^Department of Animal Science, School of Agriculture and Biology, Shanghai Jiao Tong University, Shanghai 200240, China

§Corresponding author:

Junjing Wu

jeanne1106@126.com

Xianwen Peng

pxwpal@163.com

Table S1 Gene content inside run-of-homozygosity islands.

| Chr | Gene start (bp) | Gene end (bp) | Gene name | Gene stable ID |
| --- | --- | --- | --- | --- |
| 2 | 77538967 | 77555519 | *PLPPR3* | ENSSSCG00000037432 |
| 2 | 77607187 | 77628605 | *MISP* | ENSSSCG00000013425 |
| 2 | 77619199 | 77644587 | *PALM* | ENSSSCG00000039575 |
| 2 | 77650750 | 77656013 | *PRSS57* | ENSSSCG00000020780 |
| 2 | 77656663 | 77662613 | *FSTL3* | ENSSSCG00000037102 |
| 2 | 77673323 | 77683301 | *RNF126* | ENSSSCG00000039551 |
| 2 | 77683738 | 77687282 | *FGF22* | ENSSSCG00000039236 |
| 2 | 77694253 | 77708981 | *POLRMT* | ENSSSCG00000035693 |
| 2 | 77706720 | 77730741 | *HCN2* | ENSSSCG00000033442 |
| 2 | 77753609 | 77759997 | *GZMM* | ENSSSCG00000032810 |
| 2 | 77761590 | 77768434 | *CDC34* | ENSSSCG00000039640 |
| 2 | 77773629 | 77783424 | *TPGS1* | ENSSSCG00000036434 |
| 2 | 77804685 | 77812337 | *ODF3L2* | ENSSSCG00000032811 |
| 2 | 77812415 | 77841758 | *SHC2* | ENSSSCG00000040083 |
| 2 | 77849526 | 77850788 | *C2CD4C* | ENSSSCG00000037177 |
| 2 | 77894543 | 77905959 | *THEG* | ENSSSCG00000014002 |
| 2 | 77912761 | 77938853 | *MIER2* | ENSSSCG00000014003 |
| 2 | 78091031 | 78113389 | *FLT4* | ENSSSCG00000014007 |
| 2 | 78125879 | 78127854 | *SCGB3A1* | ENSSSCG00000014008 |
| 2 | 78130842 | 78202504 | *CNOT6* | ENSSSCG00000014009 |
| 2 | 78284804 | 78330262 | *GFPT2* | ENSSSCG00000014012 |
| 2 | 78338369 | 78399632 | *MAPK9* | ENSSSCG00000014010 |
| 3 | 38387613 | 38529150 | *CREBBP* | ENSSSCG00000007951 |
| 3 | 38536671 | 38603473 | *TRAP1* | ENSSSCG00000007952 |
| 3 | 38589683 | 38646857 | *DNASE1* | ENSSSCG00000036527 |
| 3 | 38646582 | 38672494 | *SLX4* | ENSSSCG00000007954 |
| 3 | 38691242 | 38714858 | *NLRC3* | ENSSSCG00000007956 |
| 3 | 38713739 | 38753813 | *CLUAP1* | ENSSSCG00000007955 |
| 3 | 38760133 | 38761462 | *C16orf90* | ENSSSCG00000007957 |
| 3 | 38762113 | 38782056 | *NAA60* | ENSSSCG00000021065 |
| 3 | 38805243 | 38815170 | *ZNF174* | ENSSSCG00000007958 |
| 3 | 38832914 | 38833849 | *OR2C1* | ENSSSCG00000007959 |
| 3 | 38893694 | 38900332 | *TIGD7* | ENSSSCG00000037309 |
| 3 | 38905412 | 38913851 | *ZNF263* | ENSSSCG00000007963 |
| 3 | 38952346 | 38975696 | *ZNF200* | ENSSSCG00000007965 |
| 3 | 39001047 | 39001988 | *OR1F1* | ENSSSCG00000007961 |
| 3 | 39074453 | 39077929 | *ZNF213* | ENSSSCG00000007967 |
| 3 | 39087632 | 39123442 | *ZNF205* | ENSSSCG00000029375 |
| 3 | 39113402 | 39118779 | *ZSCAN10* | ENSSSCG00000029641 |
| 3 | 39130781 | 39142303 | *MMP25* | ENSSSCG00000021569 |
| 3 | 39153524 | 39162727 | *BICDL2* | ENSSSCG00000020895 |
| 3 | 39157551 | 39164625 | *THOC6* | ENSSSCG00000023315 |
| 3 | 39164375 | 39166043 | *HCFC1R1* | ENSSSCG00000027252 |
| 3 | 39166292 | 39168358 | *TNFRSF12A* | ENSSSCG00000027130 |
| 3 | 39171390 | 39172135 | *CLDN6* | ENSSSCG00000023743 |
| 3 | 39173301 | 39173954 | *CLDN9* | ENSSSCG00000021536 |
| 3 | 39201878 | 39211380 | *PKMYT1* | ENSSSCG00000029264 |
| 3 | 39206096 | 39213229 | *PAQR4* | ENSSSCG00000026248 |
| 3 | 39214251 | 39218776 | *KREMEN2* | ENSSSCG00000035052 |
| 3 | 39221752 | 39242675 | *FLYWCH1* | ENSSSCG00000028381 |
| 3 | 39251487 | 39261226 | *FLYWCH2* | ENSSSCG00000022630 |
| 3 | 39275288 | 39297969 | *SRRM2* | ENSSSCG00000023304 |
| 3 | 39302486 | 39304459 | *PRSS33* | ENSSSCG00000030518 |
| 3 | 39315423 | 39322841 | *PRSS21* | ENSSSCG00000022744 |
| 3 | 39365802 | 39376023 | *PRSS22* | ENSSSCG00000035594 |
| 3 | 39392782 | 39400042 | *PRSS27* | ENSSSCG00000008062 |
| 3 | 39429924 | 39512608 | *PDPK1* | ENSSSCG00000037719 |
| 3 | 39572531 | 39574971 | *NTN3* | ENSSSCG00000008054 |
| 3 | 39578936 | 39584364 | *TEDC2* | ENSSSCG00000008055 |
| 3 | 39586983 | 39600435 | *CCNF* | ENSSSCG00000026326 |
| 3 | 39689169 | 39735306 | *ABCA3* | ENSSSCG00000008051 |
| 3 | 39739077 | 39758379 | *RNPS1* | ENSSSCG00000008048 |
| 3 | 39750724 | 39758516 | *ECI1* | ENSSSCG00000034338 |
| 3 | 39757417 | 39766442 | *DNASE1L2* | ENSSSCG00000024587 |
| 3 | 39757736 | 39772495 | *E4F1* | ENSSSCG00000008047 |
| 3 | 39762608 | 39782525 | *PGP* | ENSSSCG00000036614 |
| 3 | 39778757 | 39786947 | *MLST8* | ENSSSCG00000008044 |
| 3 | 39781150 | 39782393 | *BRICD5* | ENSSSCG00000008045 |
| 3 | 39794208 | 39810008 | *CASKIN1* | ENSSSCG00000033059 |
| 3 | 39810108 | 39828514 | *TRAF7* | ENSSSCG00000008043 |
| 3 | 39828605 | 39837527 | *RAB26* | ENSSSCG00000033691 |
| 3 | 39829028 | 39829111 | *SNORD60* | ENSSSCG00000031232 |
| 3 | 39849512 | 39899455 | *PKD1* | ENSSSCG00000008041 |
| 3 | 39896887 | 39979949 | *TSC2* | ENSSSCG00000008040 |
| 3 | 39935674 | 39941882 | *NTHL1* | ENSSSCG00000008039 |
| 3 | 39940541 | 39952857 | *SLC9A3R2* | ENSSSCG00000008038 |
| 3 | 39962944 | 39972053 | *ZNF598* | ENSSSCG00000023400 |
| 3 | 39975074 | 39979216 | *SYNGR3* | ENSSSCG00000008036 |
| 3 | 39986152 | 39996178 | *TBL3* | ENSSSCG00000037686 |
| 3 | 39987576 | 39989530 | *NOXO1* | ENSSSCG00000008034 |
| 3 | 39997655 | 39999235 | *RNF151* | ENSSSCG00000034728 |
| 3 | 40000687 | 40000812 | *ACA64* | ENSSSCG00000036739 |
| 3 | 40001109 | 40005691 | *NDUFB10* | ENSSSCG00000030328 |
| 3 | 40002006 | 40002140 | *SNORA64* | ENSSSCG00000026828 |
| 3 | 40002631 | 40002758 | *SNORA64* | ENSSSCG00000027755 |
| 3 | 40005075 | 40016701 | *RPL3L* | ENSSSCG00000032657 |
| 3 | 40017161 | 40041393 | *HS3ST6* | ENSSSCG00000031216 |
| 3 | 40017161 | 40147930 | *MSRB1* | ENSSSCG00000032932 |
| 3 | 40092530 | 40133171 | *MEIOB* | ENSSSCG00000025711 |
| 3 | 40129381 | 40162928 | *HAGH* | ENSSSCG00000008012 |
| 3 | 40141132 | 40141806 | *FAHD1* | ENSSSCG00000039715 |
| 3 | 40174764 | 40177787 | *IGFALS* | ENSSSCG00000008013 |
| 3 | 40177994 | 40186352 | *NUBP2* | ENSSSCG00000008014 |
| 3 | 40184620 | 40194001 | *SPSB3* | ENSSSCG00000039024 |
| 3 | 40191048 | 40194049 | *EME2* | ENSSSCG00000021467 |
| 3 | 40194070 | 40196863 | *MRPS34* | ENSSSCG00000008016 |
| 3 | 40195628 | 40196869 | *NME3* | ENSSSCG00000008017 |
| 3 | 40195649 | 40241235 | *MAPK8IP3* | ENSSSCG00000008018 |
| 3 | 40244011 | 40256992 | *JPT2* | ENSSSCG00000008019 |
| 3 | 40257190 | 40296251 | *CRAMP1* | ENSSSCG00000008026 |
| 3 | 40309635 | 40373374 | *IFT140* | ENSSSCG00000008020 |
| 3 | 40343368 | 40356138 | *TMEM204* | ENSSSCG00000008021 |
| 3 | 40370755 | 40386777 | *TELO2* | ENSSSCG00000008022 |
| 3 | 40382657 | 40393901 | *PTX4* | ENSSSCG00000037262 |
| 3 | 40398114 | 40422032 | *CLCN7* | ENSSSCG00000008024 |
| 3 | 40421654 | 40430317 | *CCDC154* | ENSSSCG00000028139 |
| 3 | 40439995 | 40444934 | *C16orf91* | ENSSSCG00000038882 |
| 3 | 40510839 | 40536759 | *GNPTG* | ENSSSCG00000008031 |
| 3 | 40512959 | 40550505 | *BAIAP3* | ENSSSCG00000008029 |
| 3 | 40534222 | 40536768 | *TSR3* | ENSSSCG00000008030 |
| 3 | 40555325 | 40570053 | *UBE2I* | ENSSSCG00000021560 |
| 3 | 40710043 | 40830801 | *SOX8* | ENSSSCG00000037970 |
| 3 | 40748457 | 40749884 | *C1QTNF8* | ENSSSCG00000032400 |
| 3 | 40756457 | 40762895 | *SSTR5* | ENSSSCG00000033234 |
| 3 | 40841289 | 40929181 | *LMF1* | ENSSSCG00000008009 |
| 3 | 40950619 | 40950725 | *U6* | ENSSSCG00000031625 |
| 3 | 40982290 | 40982387 | *U6* | ENSSSCG00000020300 |
| 3 | 40996991 | 41009626 | *CHTF18* | ENSSSCG00000008010 |
| 3 | 41000297 | 41000645 | *GNG13* | ENSSSCG00000008011 |
| 3 | 41009611 | 41015540 | *RPUSD1* | ENSSSCG00000008000 |
| 3 | 41016457 | 41025590 | *MSLNL* | ENSSSCG00000023487 |
| 3 | 41042691 | 41053102 | *NARFL* | ENSSSCG00000008002 |
| 3 | 41049202 | 41053482 | *HAGHL* | ENSSSCG00000008003 |
| 3 | 41053268 | 41062032 | *FAM173A* | ENSSSCG00000008004 |
| 3 | 41062226 | 41064276 | *METRN* | ENSSSCG00000008005 |
| 3 | 41071705 | 41084077 | *FBXL16* | ENSSSCG00000008006 |
| 3 | 41085463 | 41095204 | *WDR24* | ENSSSCG00000008007 |
| 3 | 41089903 | 41095204 | *JMJD8* | ENSSSCG00000007988 |
| 3 | 41092449 | 41095187 | *STUB1* | ENSSSCG00000008008 |
| 3 | 41095840 | 41099592 | *RHBDL1* | ENSSSCG00000007998 |
| 3 | 41101356 | 41106306 | *RHOT2* | ENSSSCG00000007997 |
| 3 | 41106720 | 41122166 | *WDR90* | ENSSSCG00000007991 |
| 3 | 41123037 | 41127917 | *MCRIP2* | ENSSSCG00000007995 |
| 3 | 41134241 | 41135877 | *METTL26* | ENSSSCG00000032188 |
| 3 | 41134261 | 41138570 | *WFIKKN1* | ENSSSCG00000037013 |
| 3 | 41164401 | 41177749 | *PIGQ* | ENSSSCG00000007992 |
| 3 | 41178504 | 41182813 | *PRR35* | ENSSSCG00000007989 |
| 3 | 41190825 | 41208652 | *CAPN15* | ENSSSCG00000007993 |
| 3 | 41208946 | 41285020 | *RAB11FIP3* | ENSSSCG00000035854 |
| 3 | 41290815 | 41303988 | *DECR2* | ENSSSCG00000007987 |
| 3 | 41306175 | 41310312 | *NME4* | ENSSSCG00000007986 |
| 3 | 41317570 | 41333517 | *TMEM8A* | ENSSSCG00000007985 |
| 3 | 41323609 | 41334451 | *MRPL28* | ENSSSCG00000007984 |
| 3 | 41348117 | 41394714 | *AXIN1* | ENSSSCG00000023204 |
| 3 | 41394197 | 41398348 | *PDIA2* | ENSSSCG00000007983 |
| 3 | 41397527 | 41400086 | *ARHGDIG* | ENSSSCG00000038979 |
| 3 | 41442661 | 41473115 | *LUC7L* | ENSSSCG00000007979 |
| 3 | 78441093 | 78727940 | *WDPCP* | ENSSSCG00000032229 |
| 3 | 78895882 | 78903217 | *OTX1* | ENSSSCG00000037564 |
| 3 | 78896498 | 79345378 | *EHBP1* | ENSSSCG00000008372 |
| 3 | 79415717 | 79422393 | *TMEM17* | ENSSSCG00000026713 |
| 3 | 79671388 | 79688050 | *B3GNT2* | ENSSSCG00000008374 |
| 3 | 79768407 | 79913995 | *COMMD1* | ENSSSCG00000008376 |
| 3 | 79924549 | 79945457 | *CCT4* | ENSSSCG00000008377 |
| 3 | 79952732 | 79978587 | *FAM161A* | ENSSSCG00000008378 |
| 3 | 80254765 | 80302922 | *XPO1* | ENSSSCG00000028228 |
| 3 | 80331838 | 80587365 | *USP34* | ENSSSCG00000008382 |
| 3 | 80389466 | 80389559 | *U6* | ENSSSCG00000019479 |
| 3 | 80625899 | 80648230 | *C2orf74* | ENSSSCG00000008384 |
| 3 | 80674990 | 80746281 | *KIAA1841* | ENSSSCG00000008386 |
| 3 | 80757116 | 80791028 | *PEX13* | ENSSSCG00000008387 |
| 3 | 80872286 | 80911959 | *REL* | ENSSSCG00000008388 |
| 3 | 80989887 | 81025336 | *PAPOLG* | ENSSSCG00000008389 |
| 3 | 81197093 | 81296164 | *BCL11A* | ENSSSCG00000008392 |
| 3 | 81769739 | 81769817 | *ssc-mir-9789* | ENSSSCG00000038341 |
| 4 | 48303073 | 48303179 | *U6* | ENSSSCG00000036196 |
| 4 | 48556967 | 48862985 | *MMP16* | ENSSSCG00000006132 |
| 7 | 31222487 | 31297939 | *PPARD* | ENSSSCG00000001539 |
| 7 | 31327620 | 31330699 | *RPL10A* | ENSSSCG00000001543 |
| 7 | 31337890 | 31351452 | *TEAD3* | ENSSSCG00000001544 |
| 7 | 31362874 | 31379114 | *TULP1* | ENSSSCG00000035972 |
| 7 | 31441760 | 31585293 | *FKBP5* | ENSSSCG00000001549 |
| 7 | 31592085 | 31601773 | *ARMC12* | ENSSSCG00000001550 |
| 7 | 31618920 | 31621132 | *CLPS* | ENSSSCG00000001552 |
| 7 | 31624649 | 31632561 | *LHFPL5* | ENSSSCG00000001553 |
| 7 | 31645755 | 31701405 | *SRPK1* | ENSSSCG00000001554 |
| 7 | 31722787 | 31779819 | *SLC26A8* | ENSSSCG00000001555 |
| 7 | 31791398 | 31862031 | *MAPK14* | ENSSSCG00000001556 |
| 7 | 31880641 | 31889455 | *MAPK13* | ENSSSCG00000038774 |
| 7 | 31925546 | 31966002 | *BRPF3* | ENSSSCG00000020803 |
| 7 | 31988396 | 32025245 | *PNPLA1* | ENSSSCG00000001559 |
| 7 | 32032485 | 32054703 | *C6orf222* | ENSSSCG00000001560 |
| 7 | 32087432 | 32108185 | *ETV7* | ENSSSCG00000001561 |
| 7 | 32110530 | 32125098 | *PXT1* | ENSSSCG00000036420 |
| 7 | 32115084 | 32195072 | *KCTD20* | ENSSSCG00000001562 |
| 7 | 32206534 | 32253679 | *STK38* | ENSSSCG00000001563 |
| 7 | 32359455 | 32362271 | *CDKN1A* | ENSSSCG00000001565 |
| 7 | 32372459 | 32401798 | *RAB44* | ENSSSCG00000001566 |
| 7 | 32398573 | 32508361 | *CPNE5* | ENSSSCG00000001567 |
| 7 | 32547936 | 32584311 | *C6orf89* | ENSSSCG00000035341 |
| 7 | 32600965 | 32610739 | *PI16* | ENSSSCG00000001570 |
| 7 | 32617426 | 32635580 | *MTCH1* | ENSSSCG00000001571 |
| 7 | 32647677 | 32674541 | *FGD2* | ENSSSCG00000001572 |
| 7 | 70762126 | 70762232 | *U6* | ENSSSCG00000035242 |
| 8 | 56220528 | 56232662 | *NOA1* | ENSSSCG00000025792 |
| 8 | 56231771 | 56285011 | *POLR2B* | ENSSSCG00000022955 |
| 8 | 56279895 | 56357881 | *IGFBP7* | ENSSSCG00000008913 |
| 11 | 34192687 | 34192819 | *SNORA31* | ENSSSCG00000020435 |
| 11 | 35990414 | 35990520 | *U6* | ENSSSCG00000018929 |
